# Pharmacogenomics Guided-Personalization of Warfarin and Tamoxifen

**DOI:** 10.3390/jpm7040020

**Published:** 2017-12-13

**Authors:** Theodore J. Wigle, Laura E. Jansen, Wendy A. Teft, Richard B. Kim

**Affiliations:** 1 Schulich School of Medicine and Dentistry, Western University, London, ON N6A 5A5, Canada; twigle@uwo.ca; 2 Department of Medicine, Division of Clinical Pharmacology, Western University, London, ON N6A 5A5, Canada; laura.jansen8@gmail.com (L.E.J.); wteft@uwo.ca (W.A.T.); 3 Department of Physiology and Pharmacology, Western University, London, ON N6A 5A5, Canada; 4 Department of Oncology, Western University, London, ON N6A 5A5, Canada

**Keywords:** pharmacogenomics, warfarin, tamoxifen, cytochrome P450

## Abstract

The use of pharmacogenomics to personalize drug therapy has been a long-sought goal for warfarin and tamoxifen. However, conflicting evidence has created reason for hesitation in recommending pharmacogenomics-guided care for both drugs. This review will provide a summary of the evidence to date on the association between cytochrome P450 enzymes and the clinical end points of warfarin and tamoxifen therapy. Further, highlighting the clinical experiences that we have gained over the past ten years of running a personalized medicine program, we will offer our perspectives on the utility and the limitations of pharmacogenomics-guided care for warfarin and tamoxifen therapy.

## 1. Introduction

Warfarin and tamoxifen are widely prescribed and clinically important drugs for the treatment of conditions that require anticoagulation and estrogen receptor (ER)-positive breast cancer, respectively. The clinical response and efficacy of both drugs are variable likely due in part to genetic differences in pharmacogenes that mediate their metabolism and clearance. While genomics-based clinical guidelines have been established for several drugs, whether pharmacogenomics can be used to personalize treatment for individual patients remains controversial for warfarin and tamoxifen. This review aims to discuss the evidence to date that seek to correlate pharmacogenomic testing and the clinical outcomes in the context of each drug. Further, we will provide clinical insights and future perspectives based on our experiences with the implementation of personalized medicine strategies for warfarin and tamoxifen within a large acute care hospital setting. 

## 2. Personalizing Warfarin Therapy

Warfarin is an oral anticoagulant that is indicated for the treatment and prevention of thrombosis related complication such as stroke and pulmonary embolism among patients with atrial fibrillation, prosthetic heart valves and venous thrombosis. Warfarin is an inhibitor of vitamin K epoxide reductase complex subunit 1 (VKORC1), which prevents the cycling of vitamin K to its active metabolite. Reduction in levels of active vitamin K_1_ leads to a deficiency in many components for the coagulation cascade including factors II, VII, IX, and X. Vitamin K antagonism has proven to be difficult to manage due to marked interpatient variability of response originating from genetic, environmental and iatrogenic influences. Warfarin therapy requires frequent monitoring of the international normalized ratio (INR) of prothrombin time. Healthy individuals have an INR near 1 and the target range for the majority of warfarin-treated patients is 2–3. Out of range INR can be a warning sign of reduced medication compliance, dietary changes, and potential drug interactions that increase the risk of adverse events, where low INR fails to prevent thrombotic events and high INR results in increased risk of bleeding [1]. The adverse event rate for warfarin therapy is a major difficulty that is confronted by clinicians. For example, Budnitz et al. found that adverse drug events accounted for 2.5% of visits to American emergency departments, and that warfarin alone accounted for approximately 6% of these visits [2]. A follow up study in elderly adults over 65 years of age demonstrated that 3.6% of emergency department visits were due to adverse drug reactions, of which 17% were attributable to warfarin [3]. The dangerously high adverse event rate with warfarin usage has led to a number of studies, both in vitro and in vivo, examining the pathways and determinants that govern the observed variation in warfarin dose response, and development of more predictive dosing algorithms, including those that take into account patient-specific pharmacogenomic information.

### 2.1. Warfarin Metabolism by Cytochrome P450s

**Figure 1:**
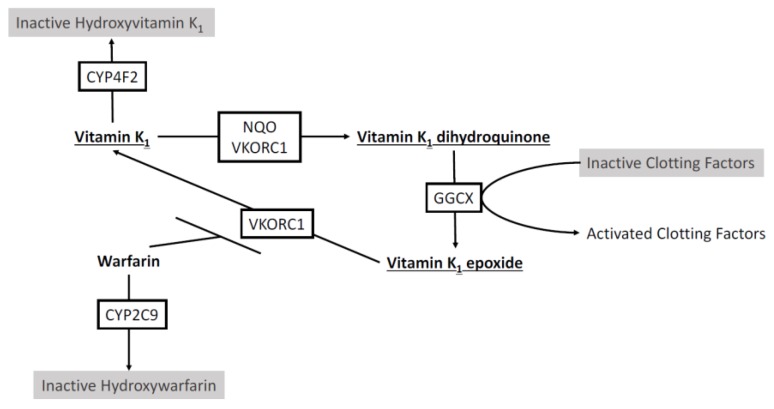
Schematic of the effect of warfarin and involvement of CYP2C9, CYP4F2, and vitamin K epoxide reductase complex subunit 1 (VKORC1) on the vitamin K cycle. VKORC1 and NAD(P)H:Quinone oxidoreductase (NQO) reduce vitamin K_1_ to active vitamin K_1_ dihydroquinone. Gamma-glutamyl carboxylase (GGCX) catalyzes carboxylation of glutamate residues activating clotting factors II, VII, IX, and X in a vitamin K_1_ dihydroquinone dependent manner. VKORC1 reduces vitamin K_1_ epoxide to vitamin K_1_, restarting the cycle. Warfarin impairs VKORC1 and the reduction of vitamin K_1_. Warfarin is metabolized by CYP2C9. Vitamin K_1_ can be removed from the cycle by CYP4F2 by hydroxylation.

Warfarin is delivered as a racemic mixture whereby the *S*-enantiomer is a significantly more potent inhibitor of VKORC1, to the extent that *S*-warfarin is believed to be the clinically relevant compound [4]. The enantiomers are metabolized by different cytochrome P450 (CYP) enzymes [5] with *S*-warfarin metabolized by CYP2C9 to the inactive *S*-7-hydroxywarfarin in the liver (Figure 1) [6]. *CYP2C9* is known to harbor common genetic variations. Specifically, *CYP2C9* variant alleles *2 and *3, are not only common, but result in an enzyme with impaired activity and decreased warfarin turnover in vitro [7,8]. CYP2C9*2 and CYP2C9*3 have been shown to have an allele frequency of 12.5% and 8.5% in Caucasian populations [8], and genotyping for these alleles has demonstrated correlation with reduced dosage requirements and a greater likelihood of over anticoagulation [9,10,11,12,13]. *CYP2C9* genotyping has been found to account for approximately 12% of the variation in warfarin dose requirement in Caucasian populations [12], leaving a large portion of the variability to be explained by other genetic factors and the environment.

Cytochrome P450 enzymes also play a direct role in the vitamin K cycle, distinct from their activity in warfarin metabolism. CYP4F2 has been shown to influence warfarin activity through its function as a vitamin K_1_ oxidase, resulting in the removal of vitamin K_1_ from the vitamin K cycle [14]. Warfarin also breaks the vitamin K cycle by impairing VKORC1 and preventing transformation to active vitamin K metabolite, therefore CYP4F2 activity increases the effect of warfarin anticoagulation (Figure 1). Caldwell et al. identified a *CYP4F2* nonsynonymous single nucleotide polymorphism (SNP) rs2108622 that results if V433M coding mutation. This polymorphism (rs2108622) was then shown to be significantly associated with increased warfarin dose requirements in a Caucasian population [15]. *CYP4F2* rs2108622 results in reduced CYP4F2 expression and activity effectively increasing the homeostatic pool of vitamin K_1_ that must be reduced for effective anticoagulation, potentially necessitating an increased dose of warfarin [14]. The rs2108622 variant of *CYP4F2* has been predicted to account for between 1% and 2% of warfarin dose variation in Caucasian populations [12,15]. As well, *CYP4F2* genotyping has demonstrated that the difference in warfarin dosing appears to occur during the induction phase of treatment [16,17]. Combining the two cytochrome P450 enzymes, CYP2C9 and CYP4F2, and their variants could predict approximately 15% of the variation in warfarin dose response in Caucasian populations.

VKORC1 is the target enzyme that is inhibited by warfarin and the rate limiting enzyme in the vitamin K cycle, allowing for warfarin to effectively prevent the production of vitamin K dependent coagulation factors (Figure 1) [18,19]. Elucidation of the gene *VKORC1* as the target for warfarin response, through the in-depth genetic assessment of patients exhibiting warfarin resistance was a crucial discovery that led to the identification of common genetic variations that predict warfarin dose and sensitivity in the general population [20,21]. Indeed, the *VKORC1* SNP rs9923231 (*2) has now been well established as an important determinant of VKORC1 response to warfarin therapy [22,23]. Genotyping of *VKORC1* demonstrates that VKORC1*2 individuals are sensitized to the effects of warfarin, and therefore require a reduced dose [24], and genotyping studies have shown that VKORC1*2 can account for over 30% of the variation in warfarin dose [25,26]. Studies combining the impact of *VKORC1*, *CYP2C9*, and *CYP4F2* variants have shown that *VKORC1* and *CYP2C9* have the most appreciable effect and account for 40% of variation in warfarin dose requirements in Caucasian populations. With up to 40% of warfarin variation in Caucasian patients accounted for through genetic polymorphisms, additional variation can be incorporated from clinical variables. Age, weight, body surface area, sex, smoking status, and indication for warfarin use have all been implicated to account for some of the variation in warfarin response [27,28]. In Caucasian populations, the combination of genetic and clinical factors predicted 57% of variation [28].

### 2.2. The Warfarin Clinical Trial Debate

The wealth of literature on the determinants of warfarin dose requirements has led to the development of a number of pharmacogenomics-guided dosing algorithms both for initiation and maintenance dosing of warfarin [28,29,30,31]. Efforts to validate pharmacogenomics based algorithms for warfarin dosing have shown that such algorithms can improve the accuracy of dose prediction (Table 1). However, converting theoretical benefit into validated clinical benefit has proven difficult, with randomized trials producing both positive and neutral results for pharmacogenomics based algorithms. Two large randomized controlled trials that were published in 2013 attempted to answer whether there was a benefit to utilizing pharmacogenetics in the dosing of warfarin [32,33]. The European Pharmacogenetics of Anticoagulant Therapy (EU-PACT) trial utilized a pharmacogenomic algorithm including clinical data, CYP2C9*2, CYP2C9*3, and VKORC1*2 genotype for the induction phase of warfarin therapy against a standard clinical dosing regimen. When comparing the primary outcome of time in therapeutic INR range and secondary outcomes of excessive anticoagulation, and time to stable INR, the EU-PACT trial demonstrated a benefit for use of pharmacogenetic algorithms during initiation of warfarin therapy [32]. However, the Clarification of Optimal Anticoagulation through Genetics (COAG) trial published in the same month also used a very similar algorithm when compared against a control group that utilized clinical data for dose prediction during induction therapy, and failed to find a statistically significant difference in the same primary outcome, time in therapeutic range [33]. A trial in Italian Caucasians also failed to detect a difference in time in therapeutic INR range when compared against a clinically guided dosing algorithm [34].

Discrepancies among clinical trials have led to many questions over the value of pharmacogenetic-guided dosing. The EU-PACT trial can be criticized for the control group lacking any standardized clinical algorithm for dosing decisions where the typical loading dose regimen may have resulted in unreasonable levels of over anticoagulation in the control arm. However, at the time of publication, the authors argue common practice did not utilize clinical algorithms and dosing with a standardized format was more translatable [32]. Meanwhile, the COAG trial has received criticism over the stratification of patients by age and indication for warfarin therapy, with implications that the atrial fibrillation and venous thromboembolism patients may respond differently to the anticoagulation effect of warfarin [35]. The EU-PACT trial used pharmacogenetic dosing from day 1 of the trial, while due to the constraints of the study design, in the COAG trial many patients received pharmacogenetics information after the first dose and some authors believe it is the earliest dosing that benefits from the input of pharmacogenetics [34,35]. Additionally, the indication for warfarin use has been implicated as a determinant factor [35] with a greater benefit predicted in atrial fibrillation [31,36]. While the EU-PACT and COAG trials differed in the proportion of patients for each indication, no significant effect was observed upon further analysis by the authors [35]. The largest difference between the EU-PACT and COAG trials is found within the patient population. The EU-PACT trial consisted of a largely homogenous population of European Caucasians, while the COAG trial was a mixed population of Caucasians and African Americans. Ethnic differences in the prevalence of CYP2C9 and VKORC1 genotypes have long been known, but these trials bring this to the center of the debate.

Compounding the difficulty in understanding the clinical value of pharmacogenetics in warfarin dosing is the recent publication of the Genetics Informatics Trial (GIFT) of warfarin to prevent deep vein thrombosis post-operatively in patients older than 65 [37]. GIFT is the largest trial to date, with 1650 patients enrolled across six centres, allowing for the use of clinical outcomes as an endpoint. GIFT found that genotype guided warfarin dosing improved the composite outcome of major bleeding, INR > 4, venous thromboembolism, or death, when compared to a clinically guided dosing algorithm. The scale of GIFT allowed for the power to detect rare events and showed a significant improvement of the primary outcome between the groups. The secondary outcome in GIFT, time in the therapeutic INR range, show a significant benefit in the genotype guided group. Interestingly, the largest benefit in the secondary outcome was observed in patients considered the high-risk subgroup, those where the genotype and clinical algorithms differed by greater than 1 mg/day. Some key differences between this and previous trials are the inclusion of CYP4F2*3 genotyping and the application of the algorithm for 11 days, twice as long as previous trials [37]. As well, GIFT enrolled patients with a single indication for warfarin therapy, thereby potentially influencing the statistical significance of the study and limiting its translation to other indications. The results of GIFT add to the debate for pharmacogenomics in warfarin dosing, while the real-world application and cost-effectiveness of these programs has yet to be tested.

### 2.3. Tailoring Pharmacogenomics-Based Warfarin Dosing Algorithms

Pharmacogenomic guided warfarin dosing algorithms continue to show promise to optimize warfarin dose selection. However, one key caveat that must be kept in mind is that much of the prospective clinical trials-based evidence has been primarily derived from Caucasian populations. Even among subjects who reside in similar parts of the world, variant allele frequencies can differ widely. For example, it has been common knowledge among clinicians that subjects of Asian descent require lower warfarin dosing on average, and Lee et al. provided evidence that 87% of their Chinese study population harbored the VKORC1*2 variant. However, within that same study, the Malay cohort carrier frequency was 65% and the Indian population had much lower rates of 12% [41]. A further review of the prevalence of *VKORC1* variants in east Asian populations found that 90% of study subjects were variant carriers, while in the rest of Asia there exists a wide range from 14% to 80% in *VKORC1* variant carrier frequency [42]. In some studies, such as the COAG trial, which enrolled a large number of African-American patients, the investigators failed to include additional SNPs in *VKORC1* and *CYP2C9* that are more prevalent and more strongly associated with warfarin dose adjustment in patients of African descent [43]. Indeed, a dosing algorithm using these two novel markers showed significant improvement on the amount of variation explained (27%) as compared to the International Warfarin Pharmacogenetics Consortium (IWPC) algorithm (16%), but overall, the power remains low and further exploration for additional genetic markers in this population is warranted [44]. The variation in allele frequencies between ethnicities impairs the translation of pharmacogenomics-based warfarin dosing algorithms, as accounting for all of the alleles may be too costly and not accounting for certain alleles may limit its utility. To effectively implement pharmacogenomics-guided dosing algorithms, clinicians may need to tailor algorithms for different ethnicities by accounting for alleles that are most prevalent within that ethnic population.

### 2.4. Insights from Clinical Implementation of Pharmacogenomics-Guided Warfarin Therapy

Since 2006, our research team has been involved in the implementation of pharmacogenomics-based personalized medicine for real-world patient care. In 2008, with the support of our hospital (London Health Sciences Centre, London Ontario Canada), we started a personalized medicine clinic that was focused on optimization of drug dosing or selection using patient-specific pharmacogenomic information. Over the past nine years we have obtained informed pharmacogenetics research consent from over 4000 patients, as part of our personalized medicine research program. We are able to incorporate clinical assessments, as well as relevant genomics-guided recommendations for drugs that are known to be affected by pharmacogenetic variation, such as, warfarin, tamoxifen, azathioprine, tacrolimus, clopidogrel, capecitabine, 5-fluorouracil, and irinotecan. Feedback from referring physicians indicates that they all value our personalized medicine-based approach to warfarin dosing. In the following sections, our perspective/opinion, based on our experience of utilizing warfarin pharmacogenomic information, both for inpatient care and in the ambulatory clinic setting is outlined.

***When and how fast to genotype*:** In our experience, carrying out warfarin pharmacogenomic testing is valuable during the initiation of warfarin therapy, or when a stable dose of warfarin has not been attained. Carrying out genotyping for patients who are already at a stable dose and adequate INR time in therapeutic range is likely unnecessary. The majority of our pharmacogenomics-guided recommendations are provided within 24 h. However, we noted that for most of the patients who are just starting warfarin therapy, one or two days of a typical standard dose (e.g., 5 mg) does not significantly alter INR trajectory even among carriers of risk alleles in *VKORC1* or *CYP2C9*.

***Timely discharge from hospital*:** As our team gained more experience with inpatient consultation requests for warfarin dosing and management, we learned the capability to predict warfarin dose based on their pharmacogenomic makeup gives far greater confidence with regards to the dose of warfarin required at the time of discharge. This is related to the fact that it typically takes 7–10 days, from initiation of warfarin to reach target INR. Frequently, in a hospitalized setting, the treating physicians worry about an unexpectedly rapid or slow rise in INR, and tend to keep such patients in the hospital longer than necessary.

***Warfarin pharmacogenomics can be used as rationale for initiating alternate treatments*:** The emergence of direct oral anticoagulants (DOACs) has meant that there is now an alternative to warfarin, particularly for the patients who require oral anticoagulation for nonvalvular atrial fibrillation or venous thromboembolism (VTE). For many patients, the cost of DOACs vs. warfarin, may be the key issue. In our experience, patients who are predicted to require low or very low warfarin dose based on their pharmacogenomic profile, tend to have more variable INRs and are less able to maintain adequate time in therapeutic range. Greater bleeding risk among variant carriers in *CYP2C9* and *VKORC1* was clearly demonstrated in the Effective Anticoagulation with Factor Xa Next Generation in Atrial Fibrillation-Thrombolysis in Myocardial Infarction 48 (ENGAGE AF-TIMI 48) clinical trial that compared edoxaban vs. warfarin [45]. Therefore, for patients in our clinic who are at greater bleeding risk during the first 90 days of therapy based on their *CYP2C9* and *VKORCI* genetics and who qualify for either warfarin or DOAC therapy, we are more likely to initiate a DOAC or switch them to a DOAC.

***Dedicated expertise and clinic for follow-up still needed*:** While pharmacogenomic assessment can provide a reasonable estimate of predicted warfarin dose, routine INR monitoring and further dose adjustment, regardless of predicted dose, is still required. Moreover, warfarin drug interactions are common, both from inhibitors of CYP2C9, those that affect gut vitamin K synthesis (e.g., antibiotics), and inducers of P450 enzymes (e.g., phenytoin, phenobarbital, carbamazepine, rifampin). In severely ill patients, caution is warranted in terms of over-reliance on pharmacogenomic-based warfarin dose as such patients tend to be quite ill, often with multi-organ dysfunction and poor oral intake. Therefore, a team or a clinic with expertise in warfarin dosing and management is highly recommended.

## 3. Personalizing Tamoxifen Therapy

Tamoxifen, a selective estrogen receptor modulator (SERM) that functions as an antagonist within the breast and reproductive organs, has been widely used to treat and prevent recurrence of ER-positive breast cancer since the 1970s [46,47,48]. While most commonly used as an adjuvant therapy, tamoxifen was the first drug approved by the US Food and Drug Administration (FDA) as a chemo-preventative agent for women at high risk for breast cancer. The benefits of long-term endocrine treatment for improved disease-free survival have been demonstrated in two large randomized controlled trials for patients with early-stage hormone receptor positive disease, shifting the paradigm of treatment strategy from 5 years to 10 years of extended tamoxifen therapy [49,50,51]. Despite a reduction in recurrence rates, there is high variability observed in response to tamoxifen. Many factors, including disease type, nodal involvement, drug adherence, concomitant medications, treatment with chemotherapy, and the presence of pharmacogenetic polymorphisms all likely contribute to variation in tamoxifen efficacy [47]. Currently, the goal of personalized tamoxifen therapy to improve breast cancer outcomes is primarily focused on understanding the contribution of pharmacogenomics to tamoxifen metabolism [52].

### 3.1. Tamoxifen Metabolism by Cytochrome P450s

Tamoxifen is a prodrug that undergoes extensive hepatic metabolism by cytochrome P450 enzymes to form primary metabolites 4-hydroxytamoxifen (4-OH-TAM) and *N*-desmethyl-tamoxifen (NDM-TAM), which are both further converted to 4-hydroxy-*N*-desmethyl-tamoxifen, better known as endoxifen [46]. It is well established that the rate limiting enzyme in tamoxifen to endoxifen bioactivation is CYP2D6, while other enzymes (CYP3A4, CYP2C9, CYP2C8, and CYP2C19) likely have a smaller contribution [46,47]. While 4-OH-TAM and endoxifen share similar affinities for ER binding, approximately 100-fold greater than tamoxifen itself, endoxifen is considered to play the lead efficacious role [53]. Circulating endoxifen concentration is on average six times greater than 4-OH-TAM, and has been shown in vitro to cause ERα degradation in a concentration dependent manner [53,54]. Endoxifen concentrations are highly variable among patients, ranging from <5 nM to >100 nM, with CYP2D6 being thought to be the main source driving this variability [55].

CYP2D6, primarily expressed in the liver, is responsible for the metabolism of approximately 25% of prescription drugs currently on the market [46]. To date, there are over 100 identified single nucleotide polymorphisms (SNPs) within *CYP2D6,* making it the most polymorphic CYP enzyme [56]. In addition, patients can carry multiple copies of *CYP2D6*. The presence of decreased (e.g., *9, *10, *17, *41) or null (e.g., *3, *4, *5, *6, *7, *8) function variants can be translated to a phenotypic classification of CYP2D6 metabolic activity as either intermediate metabolizer (IM) or poor metabolizer (PM), respectively, while patients without these variants are normal metabolizers (NM) or ultrarapid metabolizers (UM) if multiple copies of *CYP2D6* are present [46,47]. In an effort to standardize terms for clinical pharmacogenomic results, the Clinical Pharmacogenetics Implementation Consortium has redefined CYP2D6 extensive metabolizers (EM) as normal metabolizers, and as such, will be referred to as NMs within this review [57]. It is well established that CYP2D6 metabolic activity correlates with endoxifen concentration with PMs having significantly lower systemic exposure [58,59,60,61]. However, there is marked interpatient variation in endoxifen concentration that is observed within CYP2D6 phenotype groups. It is likely that some of this variation can be explained by the lack of standardization for determining CYP2D6 activity. The number of variants interrogated, their differential effect on CYP2D6 activity, and how they are combined to define phenotype can create a source of discordance. For example, using simple phenotyping, patients with *1/*41 and *41/*4 genotypes would both be categorized as IMs, while the effect of each genotype on CYP2D6 activity is considerably different. To better describe metabolic activity, many studies have used more defined phenotypic groups (e.g., NM/IM and IM/PM) or have opted to implement a CYP2D6 activity score (AS) in place of phenotype, where the AS is considered the sum of the values assigned to each *CYP2D6* allele (e.g., *1/*41 and *41/*4 would have AS of 1.5 and 0.5, respectively) [62].

The extent of variation in CYP2D6 activity due to the presence of variant alleles differs among ethnicities [63,64]. The CYP2D6 PM phenotype is more commonly observed in Caucasians due to the higher prevalence of the non-functional alleles CYP2D6*4 and *5. Gene duplications leading to genotype defined UMs and the reduced function *41 allele are most prevalent in Middle Eastern populations [63]. The high frequency (approximately 41%) of the reduced function *10 allele among Asians suggests that CYP2D6 mediated drug metabolism may be slower in this population [64]. Approximately, 35% of the allele variation observed in African and African American populations is comprised of reduced function alleles, primarily *17 and *29, respectively, with African Americans having a higher frequency of non-functional alleles [63,64]. The likelihood of yet unidentified functionally consequential variants poses a further challenge when comparing results between studies from patients of different ethnic backgrounds.

In addition to genotype, CYP2D6 activity can be affected by the co-administration of interacting medications. Selective serotonin reuptake inhibitors (SSRIs) are commonly prescribed for depression, but are also frequently used to alleviate hot flash symptoms, one of the most common side effects of tamoxifen therapy [46,65]. SSRIs, can be classified as mild, moderate, and strong inhibitors based on their demonstrated ability to inhibit the metabolic activity of CYP2D6. When taken concomitantly with tamoxifen, strong inhibitors, such as paroxetine, fluoxetine, and buproprion, can result in a dramatic reduction in the ability to form endoxifen [66] and may impact tamoxifen efficacy [67]. The use of moderate (duloxetine, sertraline) and mild (venlafaxine, desvenlafaxine, citalopram, and escitalopram) inhibitors are thought to have less of an impact on tamoxifen metabolism, however, the individual effect may vary significantly suggesting that if a SSRI is required the lowest dose of a mild inhibitor should be the preferred option to minimize the impact on CYP2D6 activity [65].

### 3.2. CYP2D6 and Tamoxifen Clinical Outcomes

The importance of CYP2D6 to tamoxifen metabolism and subsequent endoxifen formation has provided logical rationale for the hypothesis that *CYP2D6* genotype correlates with tamoxifen efficacy. If *CYP2D6* genotype correlated strongly with outcomes, then up-front genotyping tests could be offered to predict the risk of recurrence. For higher risk patients with reduced or absent CYP2D6 activity, alternate dosing or treatment strategies could be proactively offered to personalize endocrine therapy potentially improving survival outcomes. However, studies spanning the past two decades fail to provide conclusive evidence for recommending *CYP2D6* genotyping as a predictive marker of tamoxifen efficacy. While some studies have demonstrated a significant correlation, many have failed to reproduce these results (summarized in Table 2).

Early studies observed significant correlation between null and reduced CYP2D6 activity alleles and worse disease outcomes, including higher rates of recurrence [68,69,70]. Similarly, Lammers et al. found that CYP2D6 PM phenotype was associated with shorter overall survival (OS) in metastatic breast cancer patients prescribed 40 mg daily tamoxifen. This study also showed that CYP2D6 inhibitor use was an independent predictor of OS [71]. Schroth et al. conducted a large retrospective study with a median follow up period of 6.3 years, including United States (US) and German cohorts of post-menopausal women diagnosed with early breast cancer and demonstrated that patients with reduced or non-function *CYP2D6* alleles had worse disease-free survival (DFS) [72]. Further, CYP2D6 PM phenotype was found to be associated with a higher risk of disease events only in the tamoxifen arm of the Austrian Breast and Colorectal Cancer Study Group trial (ABCSG) 8. This increased risk was not observed in patients who switched to the aromatase inhibitor anastrozole after two years of tamoxifen [73]. Disease outcomes were also correlated with CYP2D6 activity when phenotype was categorized based on activity score rather than metabolizer status [74,75]. In addition, several studies have shown that homozygosity for CYP2D6 *10, which is more prevalent in Asian populations, was associated with worse DFS and recurrence-free survival (RFS) [76,77]. Kiyotani et al. conducted a study with 282 Japanese breast cancer patients receiving tamoxifen monotherapy, and showed that the presence of two variant alleles was associated with worse RFS [78]. Recently, Saladores et al. found that poor CYP2D6 activity correlated with shorter distant relapse free survival, irrespective of ethnicity [75]. CYP2D6 PM male breast cancer patients have also been shown to have a higher risk of recurrence, which remained significant when adjusted for nodal status and tumor size [79].

Retrospective data from two large double-blind trials, the Arimidex, Tamoxifen, Alone, or in Combination (ATAC) and the Breast International Group (BIG) 1–98 trial, sparked controversy by failing to validate an association between *CYP2D6* genotype and tamoxifen efficacy [80,81]. A large population based case-cohort study in the United Kingdom (UK) also failed to observe an association between the common CYP2D6*4 variant and breast cancer specific survival, however, they did note that the null CYP2D6*6 allele may affect survival in patients taking tamoxifen [82]. Other studies investigating the role of *4 and reduced function variants in patients from various ethnicities, including a recent study by Hertz et al., were unable to validate a predictive role for CYP2D6 [83,84,85,86]. Interestingly, Kiyotani et al. noted that although no association between *CYP2D6* genotype and RFS was observed in patients receiving tamoxifen-combination therapy, a significant association was shown in patients on tamoxifen monotherapy [87]. They suggest that the difference in tamoxifen regimen could explain some of the contradictions in the literature, as many studies failing to validate a role for CYP2D6 were comprised of patients on combination therapy.

Study heterogeneity resulting from varying inclusion criteria, length of treatment, concomitant medications, adherence data, measured outcomes, and DNA sources combined with non-standardized genotype classification has impeded the ability to conclusively determine the association between CYP2D6 phenotype and tamoxifen efficacy. To address heterogeneity related to use of DNA extracted from tumor infiltrated tissue, Ahern et al. conduced a quantitative bias analysis based on observed concordance rates of *CYP2D6* genotypes to examine whether call errors could bias the estimates of association. They determined that genotyping errors have a negligible effect on measured outcomes, suggesting that DNA source is unlikely to be a major contributor to study discrepancies [85]. Recently, several meta-analyses have been conducted to ascertain the benefit of *CYP2D6* genotyping [88,89,90,91,92]. Results from the International Tamoxifen Pharmacogenomics Consortium meta-analysis from studies conducted globally, suggested that CYP2D6 might indeed impact tamoxifen benefit [91]. While most of the analyses conducted to date have demonstrated that CYP2D6 variant phenotypes appear to be associated with reduced survival outcomes, the associations are based upon small, heterogeneous studies with large differences in comparator groups. As such, CYP2D6 is likely important for tamoxifen efficacy, but there remains insufficient robust evidence to support the recommendation of *CYP2D6* genotyping for personalizing tamoxifen therapy. As most studies to date have been retrospective in nature, large, well-designed prospective studies with more homogenous populations are required to fully elucidate the predictive value of CYP2D6.

### 3.3. Tamoxifen Metabolism by Other CYP P450 Enzymes

Tamoxifen metabolism is affected by additional CYP enzymes, including CYP3A4, CYP2C9, and CYP2C19, however, the overall impact of polymorphisms within these genes appears minor when compared to CYP2D6 [93,94,95]. CYP3A4 activity displays high inter-individual variability and is susceptible to drug interactions [96]. Concomitant administration of rifampicin, a CYP3A4 inducer, surprisingly resulted in the dramatic reduction in plasma endoxifen concentration [97,98]. Additionally, carriers of CYP3A4*22, thought to be a reduced function variant, have been shown to have significantly increased tamoxifen and metabolite exposure. This effect is more pronounced in CYP2D6 PMs suggesting that it may provide some functional compensation in the absence of CYP2D6 metabolic activity [60,99]. Although several studies have failed to find an association between CYP2C9 variants and tamoxifen metabolism [60,93,100], two studies have observed a significant effect of CYP2C9 activity on endoxifen concentration [61,95]. The magnitude of this effect was small and likely does not have significant impact on clinical outcomes [95]. A putative role for CYP2C19 has been controversial with some studies demonstrating higher endoxifen concentration and improved outcomes in CYP2C19*17 (increased activity variant) carriers [69,94,101,102], while other studies failed to validate these findings [100,103]. A recent retrospective analysis of the International Tamoxifen Pharmacogenomics Consortium dataset of over 2000 patients observed no effect of *CYP2C19* variants (*2, reduced function and *17) on tamoxifen outcomes, an effect that remained after accounting for *CYP2D6* genotype, concluding that *CYP2C19* genotype should likely not be considered when personalizing tamoxifen therapy [104]. The role of phase II enzymes (SULTs and UGTs) and transporters (ABCB1, ABCC2) have been also been investigated and are reviewed in [93,105].

SULTs and UGTs play a key role in the metabolism of tamoxifen by catalyzing the inactivation and subsequent elimination of tamoxifen and its metabolites. Similar to *CYP2D6*, the genes that encode for SULTs and UGTs are polymorphic with some variants impacting tamoxifen metabolism. SULT1A1 is thought to be the primary SULT enzyme that is responsible for the sulfation of endoxifen and 4-OH-TAM [105]. SULT1A1*2 is associated with reduced activity, but does not appear to correlate with endoxifen concentrations. However, carriers of *SULT1A2* variants, including SULT1A2*2 and SULT1A2*3 have been associated with higher plasma endoxifen and 4-OH-TAM concentrations [93,106]. Variants within *UGT1A4*, *UGT2B15*, and *UGT2B7* have been shown to affect glucuronidation activity [93]. A study by Romero-Lorca et al. investigated the effect of variants on the concentration of endoxifen and 4-OH-TAM glucuronidated metabolites. Patients with variants *UGT1A4^48Val^*, *UGT2B7^268Tyr^*, or with wildtype genotypes for *UGT2B17^nodel^* and *UGT2B15^523Lys^* exhibited increased concentrations of active endoxifen and 4-OH-TAM [107]. A recent follow up study by the same group observed a trend for CYP2D6 PM patients to have higher active metabolite concentrations if they were carriers of the favorable genotypes they had previously identified, suggesting that genetic variation in *SULTs* and *UGTs* may contribute to improved algorithms for predicting tamoxifen outcomes [108]. Moreover, a trend for a higher risk of recurrence was noted for patients carrying a combination of SULT1A1*2/*2:UGT2B15*1/*2 or SULT1A1*2/*2:UGT2B15*2/*2 variant alleles [83]. At present, more studies are needed to better understand the role of *SULT* and *UGT* polymorphisms on tamoxifen metabolism and efficacy.

### 3.4. Therapeutic Drug Monitoring of Tamoxifen

Amid conflicting results regarding CYP2D6 activity as a biomarker for tamoxifen efficacy, more studies are now investigating the potential for endoxifen concentration to predict outcomes. As discussed above, many factors can influence the systemic exposure of endoxifen, therefore, determining the *CYP2D6* genotype is likely not enough to predict the concentration of active metabolite. Fox et al., reported that greater than 50% of low endoxifen could not be explained by *CYP2D6* genotype or use of inhibitory medications [109]. Studies in vitro, as well as in a murine tumor growth inhibition (TGI) model, have demonstrated that the effect of endoxifen ER antagonism is concentration-dependent [61,110]. Based on pharmacokinetic-pharmacodynamic modeling, optimal TGI was predicted for patients attaining endoxifen concentrations >40 nM, while <15 nM endoxifen was predicted to achieve sub-optimal (83%) TGI [110]. A putative threshold level of endoxifen has been demonstrated by two large retrospective studies. Madlensky et al., observed that patients within the lowest quintile of endoxifen concentration (<15 nM) had higher rates of recurrence when compared to the upper four quintiles [55]. Similarly, Saladores et al., reported that patients with <14 nM endoxifen had significantly short distant relapse-free survival as compared to patients with levels >35 nM [75].

Several dose escalation studies have been designed to elevate endoxifen in CYP2D6 PMs and have consistently shown that an increased daily tamoxifen dose of 30–40 mg can significantly increase levels to be similar to NM averages, without any noted increase in adverse events [111,112,113,114,115]. Additionally, Fox et al., performed dose escalations in patients based on baseline endoxifen concentration rather than *CYP2D6* genotype. Patients with <30 nM endoxifen after eight weeks of tamoxifen on the standard dose (20 mg/day) underwent dose increases by 10 mg/day increments until target endoxifen (>30 nM) or a maximum of 60 mg/day was reached. Endoxifen levels of >15 nM were achieved in 96% of the patient cohort after dose escalation when compared to 76% at baseline, with 76% of patients attaining >30 nM endoxifen [109]. This study demonstrates the potential utility of personalizing tamoxifen therapy to improve outcomes through therapeutic drug monitoring (TDM) of endoxifen. Patients with sub-therapeutic endoxifen exposure or poor adherence can be quickly identified within the first 4–8 weeks after tamoxifen initiation, providing early rationale for clinicians to continue with tamoxifen therapy or switch to an alternate treatment plan. The use of TDM may be limited in some clinical settings, therefore the development of algorithms to predict endoxifen concentration based on clinical, environmental and genetic factors are warranted. An early algorithm developed by our team based on a patient cohort of approximately 200 patients, achieved a predictive accuracy of 89%, with a cross validation estimated accuracy of 85% [60]. Larger prospective trials are needed to further develop highly predictive models and confirm the utility of TDM for personalizing tamoxifen therapy. The undertaking of such lengthy prospective trials may in fact be unrealistic due to the large body of conflicting evidence to date. However, the possibility of replacing tamoxifen with endoxifen therapy is on the horizon, as the first phase I trial results have demonstrated acceptable toxicity with promising efficacy in patients with endocrine-refractory, metastatic breast cancer [116].

### 3.5. Clinical Perspectives Based on Experience from a Personalized Tamoxifen Clinic 

Clinical application of *CYP2D6* genotyping as a way of predicting response to tamoxifen therapy has not been widely accepted. Over the past seven years, as a part of our personalized medicine research program, we have carried out *CYP2D6* genotyping, and TDM measuring both tamoxifen and endoxifen plasma concentrations using a LC-MS/MS system in approximately 800 patients. Shown below are key observations from our clinic.

***CYP2D6 genotyping alone is inadequate*:** We have observed that nearly 5% of patients, primarily of Caucasian descent, are CYP2D6 PMs. However, only a portion (60%) of those predicted to be PMs have endoxifen concentrations below 15 nM. Additionally, nearly 20% of patients predicted to be CYP2D6 IMs were observed to have endoxifen concentrations of less than 15 nM. Current clinical standard does not require or suggest tamoxifen dosing be changed based on *CYP2D6* genotype. However, we observed that a portion of patients are likely receiving sub-optimal benefit from tamoxifen and patients with low endoxifen may benefit from higher daily tamoxifen doses.

***Endoxifen measurement provides reassurance for patients regarding adequacy of tamoxifen metabolism*:** In our clinic, we often hear from our patients that they experience little to no side effects from tamoxifen, and are thus very concerned that they may not be attaining benefit from tamoxifen therapy. Since our team not only carries out *CYP2D6* genotyping, but also measures endoxifen plasma concentration, we are able to demonstrate to our patients that in fact, presence or absence of side effects cannot be used as a predictor of adequate tamoxifen metabolism.

***Tamoxifen and endoxifen measurement and patient compliance*:** Tamoxifen’s half-life is nearly seven days, thus major changes in tamoxifen and endoxifen concentrations are reflective of the overall tamoxifen compliance in the previous month prior to endoxifen measurement. Thus, when we observe markedly lower than expected tamoxifen and endoxifen concentrations, we are able to discuss the importance of medication compliance. Interestingly, our patients who are provided with their own endoxifen metabolism data, demonstrate a greater willingness to continue tamoxifen. This holds true even when patients experience significant side effects such as hot flashes, as they feel more reassured regarding the adequacy of their tamoxifen metabolism, and the likelihood of long-term clinical benefit.

***Endoxifen measurement is useful in quantifying the extent of tamoxifen drug interactions*:** One of most common and clinically impactful roles of our tamoxifen clinic relates to our ability to quantify tamoxifen drug interactions. A significant proportion of our tamoxifen patients are on antidepressants. Low doses of antidepressants are often prescribed by oncologists to reduce hot flash symptoms. Most antidepressants, particularly those in the SSRI class, act as inhibitors of CYP2D6 activity. SSRIs, such as paroxetine, fluoxetine, and bupropion are well known potent inhibitors of CYP2D6. In fact, the product monograph of such SSRIs cautions an interaction with tamoxifen that may lead to reduced endoxifen formation. By measuring a patient’s endoxifen concentration, we can demonstrate the net effect of such interactions. For patients with a low endoxifen concentration who are taking potent CYP2D6 inhibitors, we are able to document a two-fold increase in endoxifen concentration when such patients discontinue the SSRI or are switched to SSRIs with low to moderate CYP2D6 inhibitory effect [60]. We note that patients who are CYP2D6 NMs or UMs tend to exhibit endoxifen concentration above 15 nM, even when taking potent CYP2D6 inhibitors. Such information can be useful in some cases where the patient is unwilling to switch to an alternative antidepressant. Conversely, we have been able to document the deleterious effect of CYP metabolism inducing drugs, such as phenytoin and rifampin, to a reduction in both tamoxifen and endoxifen plasma concentrations [98,117].

## 4. Conclusions

Genetic variation in CYP enzymes is increasingly recognized as clinically important. Although the relationship between molecular basis of genetic variation in CYP enzymes and their expression and function is well established, the application using such information to guide relevant drug therapy in real-world patients has taken longer than expected. Much of the delay has been related to the lack of randomized clinical trials data, as well as the cost-effectiveness of pharmacogenomics-based approach. While current evidence may not support the implementation of pharmacogenomics testing that may be impractical or unfeasible in certain clinical settings, the use of available genetic information should be utilized to optimize drug therapy. Clinical guidelines have been established for warfarin dosing in patients with known genetic information [118], and guidelines for tamoxifen are in currently in development.

For warfarin, it is now becoming clear that preemptive genotyping can aid in better dosing decisions, and the most recent large scale randomized trials data suggest genotype guided approach results in better outcome. For medications such as tamoxifen, we are still awaiting decisive large scale clinical outcomes data, in terms of optimal genotype or endoxifen-based dosing. Given that tamoxifen is usually only a part of overall breast cancer treatment strategy (surgery, chemotherapy, radiation therapy), it will likely require a very large sample size and a long follow-up period to provide such conclusive evidence. What is becoming clear is that endoxifen is the major active metabolite, and the observed endoxifen level could be viewed as a surrogate marker of adequate tamoxifen dosing, and for mitigation of potentially deleterious drug interactions. The recently completed Phase I study using endoxifen suggest that there is the potential for endoxifen therapy in the future, potentially bypassing the current concerns relating to CYP2D6 and tamoxifen [116].

## Figures and Tables

**Table 1:** Association of pharmacogenomics with warfarin outcomes.

Studies	Design	*N*	Population	Alleles	Outcomes	*P*
Positive association						
Primohamed et al., 2013 [32]	RCT, genotype guided vs. standard dose	455	98% White 1% Black 1% Asian	CYP2C9*2 CYP2C9*3 VKORC1*2	Improved time within therapeutic INR (67.4% vs. 60.3%); reduction in INR > 4, reduced time to therapeutic INR	<0.001
Gage et al., 2017 [37]	RCT, genotype guided vs. clinical	1597	91% White 6% Black 2% Asian 1% Other	CYP2C9*2 CYP2C9*3 VKORC1*2 CYP4F2*3	Reduced composite measure of major bleeding, INR > 4, death, and VTE (10.8% vs. 14.7%). In hip and knee arthroplasty patients	<0.02
Caraco et al., 2008 [38]	RCT, Genotype vs. clinical	191	Unavailable	CYP2C9*2 CYP2C9*3	Reduction in time to first therapeutic INR (2.73 days earlier) and reduction in time to stable INR (18.1 days earlier)	<0.001
Gage et al., 2008 [28]	Validation of dosing algorithm	292	93% Caucasian 15% Black 2% Hispanic	CYP2C9*2 CYP2C9*3 VKORC1*2	Pharmacogenomic dose prediction more accurate than clinical dose prediction (53% vs. 17% of explained variability, respectively)	<0.0001
IWPC, 2009 [29]	Validation of dosing algorithm	1009	55% White 30% Asian 10% Black 5% Other	CYP2C9*2 CYP2C9*3 VKORC1*2 ^#^	Pharmacogenomic dose prediction more accurate than clinical dose prediction (accurately identified 49.4% vs. 33.3% of patients requiring ≤21 mg warfarin per week, respectively)	<0.001
Gong et al., 2011 [31]	Validation of dosing algorithm	167	95% White 2% Black 2%Asian 1% Other	CYP2C9*2 CYP2C9*3 VKORC1*2 CYP4F2*3	Demonstrated the safe effective prediction of dose limiting variation	N/A
Negative association						
Kimmel et al., 2013 [33]	RCT, Genotype guided vs. clinical	1015	66%White 27% Black 7% Hispanic	CYP2C9*2 CYP2C9*3 VKORC1*2	No difference in time in therapeutic INR (45.2% vs. 45.4%) No difference in anticoagulation control or dose prediction	0.91
Verhoef et al., 2013 [39]	RCT, Genotype guided vs. clinical	1597	98% White	CYP2C9*2 CYP2C9*3 VKORC1*2	No difference in time in therapeutic INR range (61.6% vs. 60.2%)	0.47
Pengo et al., 2015 [34]	RCT, Genotype guided vs. standard	180	100% White	CYP2C9*2 CYP2C9*3 VKORC1*2 CYP4F2*3	No difference in out of range INRs (45.6% vs. 43.6%) or time in therapeutic INR range (51.9% vs. 53.3%)	0.79 0.71
Anderson et al., 2007 [40]	RCT, Genotype guided vs. standard	200	94% White	CYP2C9*2 CYP2C9*3 VKORC1*2	No difference in time in therapeutic INR range (30.7% vs. 33.1%)	0.47

RCT, randomized control trial; INR, International normalization ratio; VTE, venous thromboembolism; #, VKORC1*2 or one of six other linked SNPs, N/A, not available.

**Table 2:** Association of *CYP2D6* pharmacogenomics with tamoxifen outcomes.

Studies	*N*	Alleles	DNA Source	Conclusions	Outcome	HR (95% CI)	*P*
Positive association							
Goetz et al., 2005 [68]	190	*4	PE-tissue, buccal swabs	*4/*4 patients had worse RFS and DFS	RFS DFS	2.71 (1.15–6.41) 2.44 (1.22–4.90)	0.023 0.012
Schroth et al., 2007 [69]	206	*4, *5, *10, *41, *CNV*	normal breast tissue	Decreased function alleles (*4, *5, *10 and *41) were associated with higher rates of recurrence and shorter relapse free periods	RFS EFS	2.24 (1.16–4.33) 1.89 (1.10–3.25)	0.02 0.02
Ramón et al., 2010 [70]	91	33 alleles	blood	Patients with *4/*4, *4/*41, *1/*5 or *2/*5 genotypes had shorter DFS			0.016
Lammers et al., 2010 [71]	99	*3, *4, *5, *6, *10, *41	blood	PMs had worse overall survival compared to NMs	OS	2.09 (1.06–4.12)	0.034
Schroth et al., 2009 [72]	1325	*3,*4, *5, *10, *41	blood, fresh frozen or PE-tissue	Decreased activity (NM/IM; PM) had worse EFS and DFS	EFS DFS	1.35 (1.08–1.68) 1.31 (1.06–1.61)	0.007 0.02
Goetz et al., 2013 [73]	453	*3, *4, *6, *10, *41	PE- tissue	PM/PM patients had higher risk of disease event compared to NM/NM patients	OR	2.45 (1.05–5.73	0.04
Damodaran et al., 2012 [74]	132	*1, *2, *4, *5, *10	blood	CYP2D6 activity scores <0.5 had worse RFS compared to activity scores >1	RFS	7.29 (2.92–18.2)	<0.001
Saladores et al., 2015 [75]	587	*3, *4, *5, *6, *9, *10, *41, *CNV*	blood	Improved DRFS was associated with increased CYP2D6 activity score	DRFS	0.62 (0.43–0.9)	0.013
Xu et al., 2008 [76]	152	*10	blood, fresh frozen or PE-tissue	*10/*10 was associated with worse DFS	DFS	4.7 (1.1–20.0)	0.04
Kiyotani et al., 2008 [77]	67	*4, *5, *6, *10, *14, *18, *21, *41	blood	*10/*10 genotype had worse RFS	RFS	10.04 (1.17–86.3)	0.036
Kiyotani et al., 2010 [78]	282	*4, *5, *6, *10, *14B, *18, *21, *36, *41, *CNV*	blood	Presence of two variant alleles was associated with worse RFS compared to patients with no variants	RFS	9.52 (2.79–32.45)	<0.0001
Negative association							
Rae et al., 2012 [80]	588	*2, *3, *4, *6, *10, *41	PE-tissue	PMs did not have reduced recurrence rates compared to NMs	RFS	0.99 (0.48–2.08)	0.99
Regan et al., 2012 [81]	973	*2, *3, *4, *5, *6, *7, *10, *17, *41	PE-tissue	IMs and PMs treated with tamoxifen monotherapy were not associated with BCFI	BCFI	0.86 (0.6–1.24)	0.35
Abraham et al., 2010 [82]	3155	*4, *5, *6, *9, *10, *41, *CNV*	blood	PM/IM patients did not have reduced survival outcomes compared to NMs	BCSS	0.93 (0.55–1.57)	0.78
Nowell et al., 2005 [83]	160	*3, *4, *6	PE-tissue	*4/*4, *1/*4 were not associated with reduced DFS compared to *1/*1	DFS	0.67 (0.33–1.35)	0.19
Park et al., 2012 [84]	716	*2, *5, *10, *41	blood	Homozygous variant carriers did not have reduced RFS	RFS	1.14 (0.68–1.92)	0.61
Hertz et al., 2017 [86]	476	*2, *3, *4, *6, *10, *41, *CNV*	Fresh frozen tumors	CYP2D6 activity score was not associated with RFS	RFS	1.16 (0.84–1.62)	0.37
Kiyotani et al., 2010 [87]	167	*1, *4, *5, *10, *21, *36, *41	blood	No association between genotype and RFS in patients on tamoxifen-combined therapy	RFS	0.64 (0.20–1.99)	0.44

HR, Hazard ratio; CI, confidence interval; PE, paraffin-embedded; RFS, recurrence free survival; DFS, disease free survival; CNV, copy number variation; EFS, event free survival; NM, CYP2D6 normal metabolizer; OS, overall survival; IM, CYP2D6 intermediate metabolizer; PM, CYP2D6 poor metabolizer; DRFS, distant relapse free survival; BCFI, breast cancer-free interval; BCSS, breast cancer specific survival.
